# Effects of early postnatal hyperoxia exposure combined with early ovalbumin sensitization on lung inflammation and bacterial flora in a juvenile mouse model of asthma

**DOI:** 10.3389/fmicb.2023.1220042

**Published:** 2023-07-06

**Authors:** Jingyan Li, Tianping Bao, Linxia Cao, Mengmeng Ma, Yuan Zhang, Zhaofang Tian

**Affiliations:** Department of Neonatology, The Affiliated Huaian No. 1 People’s Hospital of Nanjing Medical University, Huai’an, Jiangsu, China

**Keywords:** asthma, hyperoxia, lung microbiota, newborn, mice

## Abstract

**Objective:**

The aim of this study is to explore the effects of early postnatal hyperoxia exposure combined with early ovalbumin (OVA) sensitization on lung inflammation and bacterial flora in neonatal mice on a juvenile mouse model of asthma.

**Methods:**

Thirty-two newborn female C57BL/6 J mice were randomly divided into four groups, which including room air+phosphate-buffered saline (PBS) group, hyperoxia+PBS group, room air+OVA group, and hyperoxia+OVA group, according to the hyperoxia exposure and/or OVA induction. Mice were exposed to either 95% O_2_ or room air for 7 days after birth; after 7 days, they were exposed to air and received an intraperitoneal injection of OVA suspension or PBS solution on postnatal days 21 (P21) and 28 (P28). From P36 to P42, the mice were allowed to inhale of 1% OVA or 0.9% NaCl solution. The mice were observed after the last excitation. HE staining was performed to observe the pathological changes in lung tissues. Wright-Giemsa staining was used to perform bronchoalveolar lavage fluid (BALF) leukocyte sorting. Enzyme-linked immunosorbent assay was used to determined the cytokines levels of interleukin (IL)-2, IL-5, IL-13, IL-17A, and IL-10 and serum IgE levels in BALF. Additionally, 16S rRNA sequencing was used to analyze the characteristics of lung microbiota.

**Results:**

Mice in the hyperoxia+OVA group showed asthma-like symptoms. HE staining results revealed a significant thickening of the airway wall and airway inflammation. BALF analysis of cellular components showed significant increases in total leukocyte and eosinophil counts and the levels of cytokines related to Th2 (IL-5 and IL-13) and Th17 (IL-17A); 16S rRNA sequencing revealed that the main members of the pulmonary microflora were Actinobacteriota, Proteobacteria, Firmicutes, and Bacteroidota at the phylum level. In addition, the bacteria with a major role were Acinetobacter and Moraxellaceae in the O_2_ + OVA group.

**Conclusion:**

The mouse suffering from postnatal hyperoxia exposure and early OVA sensitization, changes in symptoms, pathology, leukocyte and eosinophil counts, and levels of different T-cell cytokines in BALF and lung microbiota, which may provide a basis for the establishment of a juvenile mouse model of asthma.

## 1. Introduction

Asthma is a heterogeneous disease characterized by airway hyperresponsiveness (AHR), airway inflammation, and reversible airway remodeling and is one of the most common chronic respiratory diseases occurring in children (Akar-Ghibril et al., 2020). Persistent asthma in children leads to inadequate lung function and increases the risk of chronic obstructive pulmonary disease and persistent airflow obstruction to the lungs in adulthood; it can also lead to sudden death in patients with severe acute attacks (Asher et al., 2021). The natural disease course of asthma is variable, and different risk factors are present at different stages of the disease ranging from fetal life to adulthood (Sánchez-García et al., 2020). Epidemiological observations and studies on immunology and lung development suggest that early life exposures and birth status have a significant effect on lung function in adulthood (Mahmoud et al., 2023). For instance, prematurity, abnormal bronchopulmonary development, and low birth weight are associated with reduced lung function in adulthood (Di Filippo et al., 2022). Bronchopulmonary dysplasia (BPD) and asthma have similar respiratory features such as bronchial hyperresponsiveness and persistent airflow limitation (Clemm et al., 2018). Infants with moderate-to-severe BPD may have a prolonged pulmonary impairment in preterm BPD that may then persist in adulthood, leading to asthma development (Gough et al., 2014). Since the 1980s, the incidence of BPD has shown an increase, especially as neonatal monitoring and the survival of extremely preterm infants have improved. BPD in preterm infants may be a major risk factor for the future development of asthma in childhood (Sun et al., 2023). Therefore, the use of early hyperoxia exposure in neonatal mice (BPD model) combined with early ovalbumin (OVA) sensitization can help in understanding the relationship between late childhood BPD and asthma development and formulating effective treatments.

Recently, increasing interest is being paid to the relationship between changes in the human microbiota and asthma development, with the “hygiene hypothesis” being the first theory to propose a link between exposure to microorganisms and allergic diseases (Strachan, 1989). In the internal environment of an organism, several microbial communities live in symbiosis with the host. However, less attention has been paid to changes in the lung flora because of the traditional view that healthy human lungs are sterile (free from bacteria) as well as the difficulty in sampling from the lower respiratory tract (Natalini et al., 2023). With advances in high-throughput sequencing technology, DNA sequencing methods have been used to identify unique bacterial flora present in healthy human lungs. The main respiratory bacterial phyla are Proteobacteria, Firmicutes, Bacteroidetes, and Actinobacteria (Hou et al., 2022). Pulmonary bacterial flora plays an important role in the development, regulation, and maintenance of immunity (Whiteside et al., 2021). When mice were exposed to microorganisms, their pulmonary inflammatory responses were exacerbated; newborn animals tended to show a strong response to allergens, and regulatory T cells appeared as the bacterial load increased, and the response to allergens was subsequently attenuated (Herbst et al., 2011; Gollwitzer et al., 2014). In an analysis of sputum and bronchoalveolar lavage fluid (BALF) samples collected from patients with severe asthma, the abundance of Proteobacteria, especially *Haemophilus* and *Moraxella*, was elevated in patients with neutrophilic asthma and was strongly associated with AHR and airway inflammation (Taylor et al., 2017; Marathe et al., 2022), suggesting the role of the respiratory microbiome in asthma pathogenesis and control.

In our previous study, we subjected mice to early postnatal hyperoxia exposure and OVA sensitization (6 weeks) and found that the airway inflammatory response had exacerbated with significant airway structural remodeling, but the experimental endpoint was 9 weeks postnatally, which represented the adulthood phase of mice (Wang et al., 2021). On the basis of a previous study, after early hyperoxia exposure, the OVA induction was advanced to 3 weeks after birth, and the study endpoint was 6 weeks after birth, which was in the adolescence of mice. This study investigated the effects of the early combined intervention on changes in lung inflammation and pulmonary bacterial flora in mice and explored the feasibility of establishing a juvenile mouse model of asthma.

## 2. Materials and methods

### 2.1. Animals

We purchased 32 female neonatal C57BL/6 J mice (SPF grade) with a birth time difference of <30 min from The Medical Animal Experiment Center of Nanjing Medical University. The mice were raised in the Animal Experiment Center of Affiliated Huaian No. 1 People’s Hospital of Nanjing Medical University. We included female mice to exclude gender differences. The mice were raised at a constant temperature of 24 ± 2°C, 60–70% relative humidity, and day and night were maintained for 12 h each. The mice were allowed free access to food and water, provided by the animal center. The study was approved by the Ethics Committee of the Affiliated Huaian No.1 People’s Hospital of Nanjing Medical University (DW-P-2021-002-01).

### 2.2. Reagents

Grade III and V OVA were purchased from Sigma (MO, United States); Al (OH)3 was acquired from Thermo Scientific Company. IgE, IL-2, IL-5, IL-13, IL-17A, and IL-10, were detected using ELISA kits. The ELISA kits were purchased from NeoBioscien Company, and the experimental steps were strictly in accordance with the kit instructions.

### 2.3. Experimental procedure

**Figure 1:**
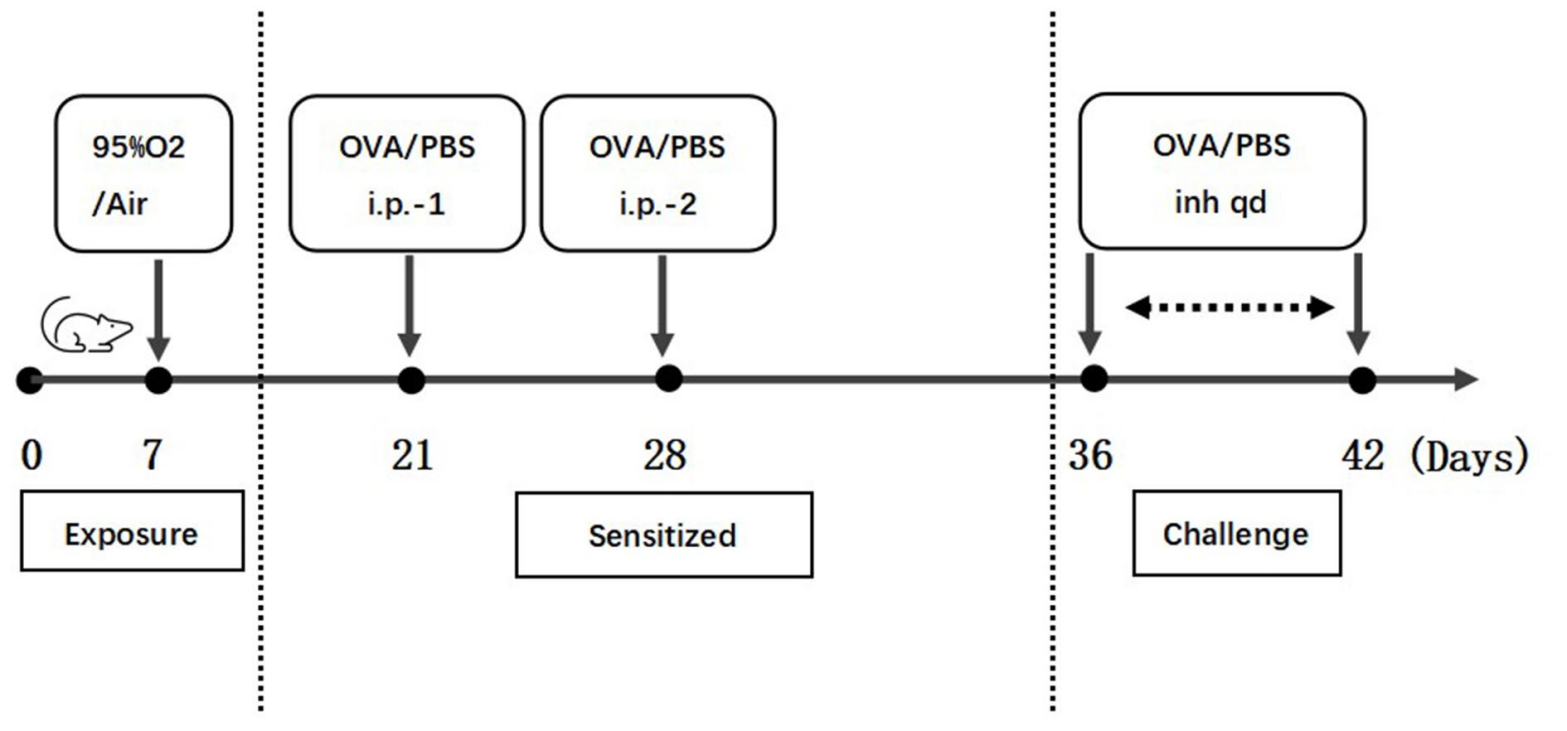
Flowchart of mouse model establishment.

Thirty-two female C57BL/6 J newborn mice were randomly divided into the following four groups: room air (RA) + phosphate-buffered saline (PBS) group, hyperoxia (O_2_) + PBS group, RA + OVA group, and O_2_ + OVA group, with eight mice in each group. The mice were exposed to either hyperoxia (95% O_2_) or room air from postnatal day 1 (P1) to P7 in sealed plexiglass chambers under continuous oxygen monitoring. Nursing dams were rotated every 24 h between the RA and O_2_ groups. The mice received an intraperitoneal injection of 100 μL of sensitization solution (OVA 1 mg/mL + Al (OH)_3_ 1 mg/mL) or an equal amount of PBS at P21 and P28. Mice were sensitized by nebulized inhalation of 1% OVA or an equal amount of PBS from P36 to P42 once daily. Within 48 h of the last challenge, the activity performance of the mice was observed. All mice were euthanized by asphyxiation with CO_2_ on P44. Figure 1 shows a flowchart of mouse model establishment.

### 2.4. Serum IgE levels

The blood was collgected from the heart, placed it in an Eppendorf tube, and allowed it to stand for 1 h at room temperature. The supernatant was collected and centrifuged at 4°C for 10 min at 4000 r/min and frozen at −80°C for further.

### 2.5. Leukocyte classification and cytokine levels In BALF samples

BALF was collected by lavage of the trachea, lungs, and bronchi. For this, 1 mL sterile saline in a 1 mL syringe pump was slowly pushed into the physiological saline. After holding for a few moments, the syringe was slowly pulled back and the lung tissue was gently pressed to collect the lavage fluid. The lavage was repeated thrice to ensure the recovery rate was >80%. The lavage obtained was centrifuged at 4000 r/min for 10 min. The precipitate was immediately counted for cell sorting, while the supernatant was frozen at −80°C for cytokine detection. Leukocytes were classified by Wright-Giemsa staining.

### 2.6. Histopathological examination

The lungs and trachea of mice were removed, visualized with the naked eye, and then rinsed with PBS. The upper lobe of the right lung was fixed in 4% paraformaldehyde, after which it was paraffin-embedded and cut into serial sections of 4 mm thickness for histopathological examination. The remaining lung tissue was immediately frozen in liquid nitrogen and transferred to a − 80°C refrigerator for storage. After histopathological examination, the hematoxylin–eosin–stained sections were evaluated for changes in airway thickness and inflammatory cell infiltrate under bright-field illumination, and images were captured. The pathology in each group was graded and scored as follows (Henderson et al., 2002; Fang et al., 2021): 0, no inflammatory cells; 1, a few inflammatory cells; 2, more uneven distribution of inflammatory cells, with a layer thickness of one cell; 3, a large number of evenly distributed inflammatory cells; and 4, a large number of inflammatory cells gathered as a mass, with a layer thickness of more than four cells. Eight bronchioles were analyzed on each tissue slide, and mean scores were calculated.

### 2.7. Genomic DNA extraction

To characterize the pulmonary microbiota, 16S rRNA sequencing was performed using lung tissue specimens collected from mice with asthma. Genomic DNA of the microbial community was extracted from samples using the E.Z.N.A.^®^ Tissue DNA Kit (Omega Bio-Tek, Norcross, GA, United States), according to the manufacturer’s instructions. The extracted DNA was run on a 1% agarose gel to separate and confirm the target band, and DNA concentration and purity were determined using the NanoDrop 2000 ultraviolet–visible spectrophotometer (Thermo Fisher Scientific, Wilmington, NC, United States). The hypervariable region V3-V4 of the bacterial 16S rRNA gene was amplified with primer pairs 338F (5′-ACTCCTACGGGAGGCAGCAG-3′) and 806R (5′-GGACTACHVGGGTWTCTAAT-3′) using the GeneAmp® 9,700 polymerase chain reaction thermocycler (ABI, CA, United States). The 16S rRNA assay was performed by Nanjing Fengzi Biomedical Technology Co., Ltd.

### 2.8. Illumina MiSeq sequencing

Purified amplicons were pooled at an equimolar ratio and paired-end sequenced on the Illumina MiSeq PE300 platform (Illumina, San Diego, CA, United States). Raw reads were deposited into the NCBI Sequence Read Archive database. Sequences were clustered into operational taxonomic units (OTUs) based on intersequence similarity by splicing, quality control, and filtering of sequenced reads with 97% consistency. Then, species annotation was performed on the obtained OTU sequences with reference to the SILVA138 database. Based on the species annotation results, beta diversity and species abundance were further analyzed.

### 2.9. Statistical analysis

SPSS 21.0 statistical software was used for statistical analysis of data. Data were presented as mean ± standard deviation. Differences among more than two groups were assessed using a one-way analysis of variance followed by the least significant difference test. Statistical significance was determined at *p* < 0.05 (^*^) or *p* < 0.01 (^**^) and nonsignificance at *p* > 0.05.

## 3. Results

### 3.1. Performance of mice

**Figure 2:**
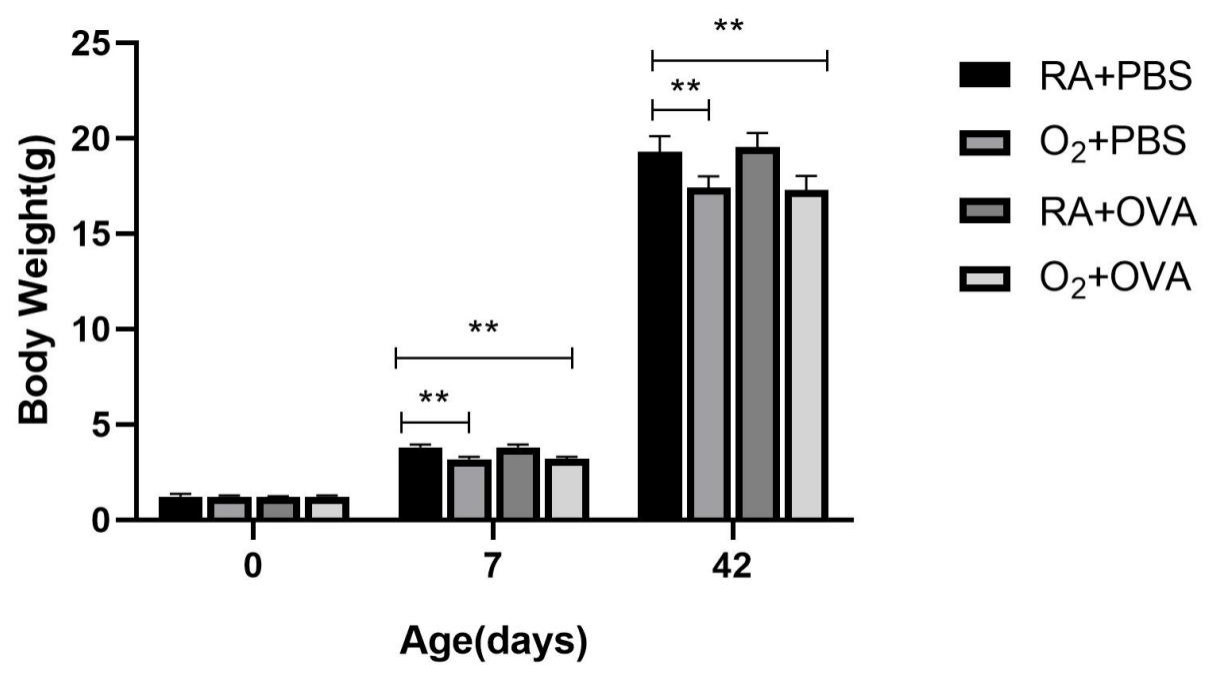
Changes in the body weight of mice (*n* = 8). ^*^p ≤ 0.05, ^**^p ≤ 0.01.

Mice in the O_2_ + PBS and O_2_ + OVA groups had a smaller body size (Figure 2), disorganized fur, and lethargy after hyperoxia exposure. Additionally, mice in the O_2_ + OVA group developed asthma-like symptoms such as ear-scratching, restlessness, arched back, and curled body after OVA sensitization.

### 3.2. Pathological examination

**Figure 3:**
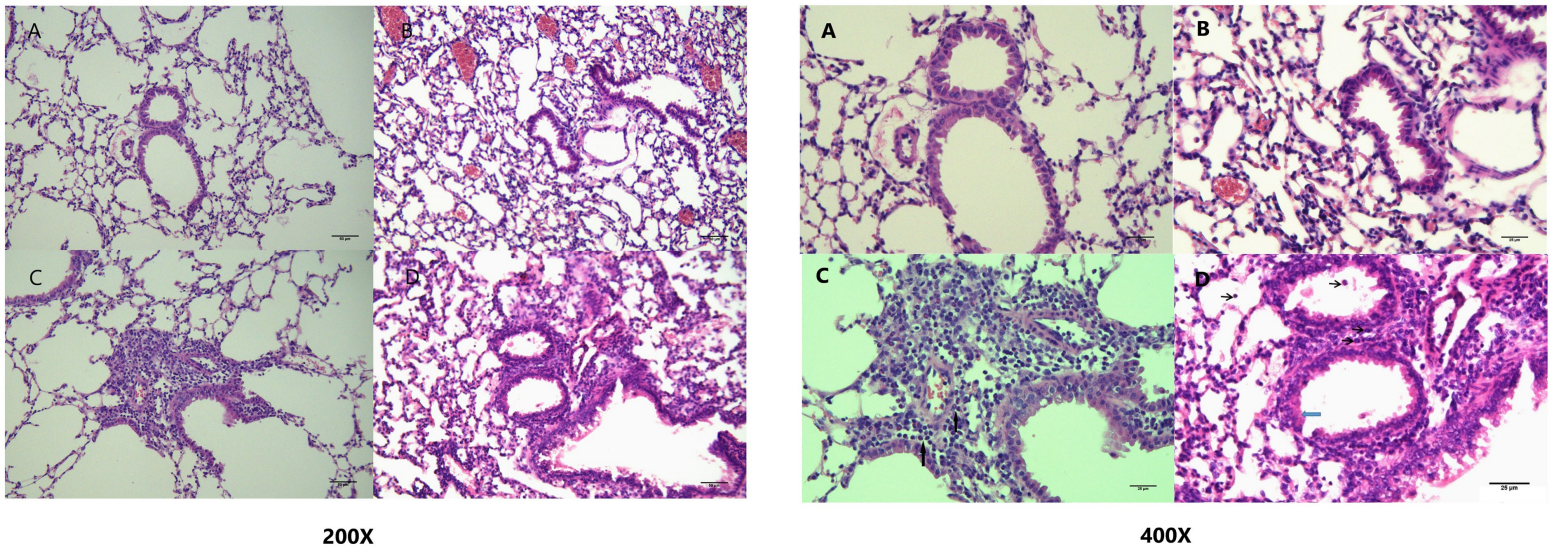
Bronchial pathological changes. (H&E staining 200× magnification; scale bar = 50 μm; 400× magnification; scale bar = 25 μm). **(A)** RA + PBS group: the tracheal structure appears normal; **(B)** O_2_ + PBS group: the airway wall is slightly thickened; **(C)** RA + OVA group: the airway columnar epithelium is enlarged, but the airway wall thickness does not show a significant increase; **(D)** O_2_ + OVA group: the airway wall is significantly thickened, together with evident lumen stenosis and hypertrophy of airway columnar epithelium. (The black arrows point to infiltrated eosinophils, and the blue arrow points to goblet cells.) H&E, hematoxylin–eosin; OVA, ovalbumin; PBS, phosphate-buffered saline.

**Figure 4:**
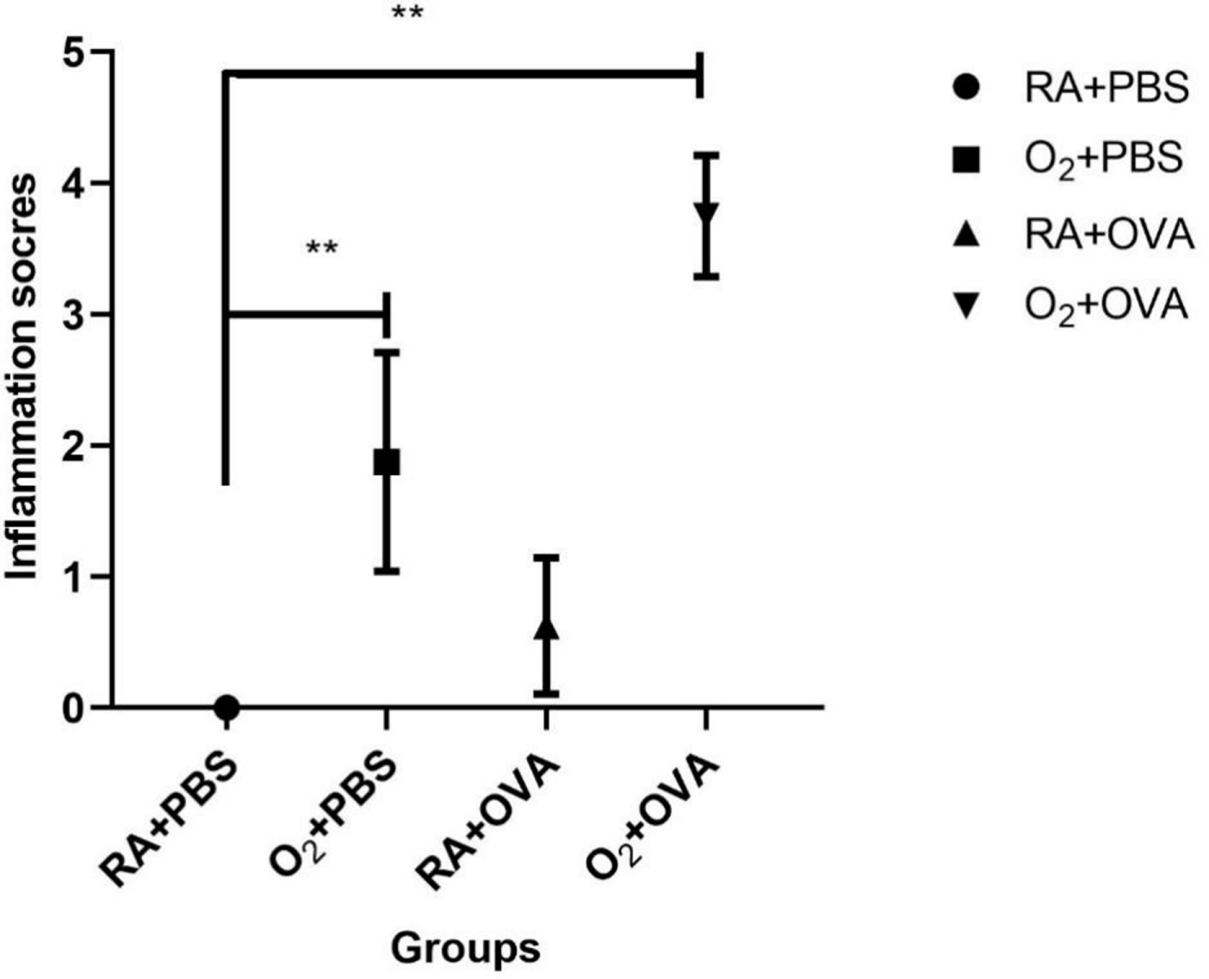
Lung inflammation scores in different groups (^**^p ≤ 0.01).

H&E staining of the lung sections revealed inflammation in the small airways and alveolar regions. The airway structure appeared normal in the RA + PBS group, whereas the airway wall was slightly thickened in the O_2_ + PBS group. Airway columnar epithelium hypertrophy was observed in the RA + OVA group, but no significant increase in airway wall thickness was observed. The airway wall was significantly thickened in the O_2_ + OVA group, together with significant luminal stenosis and airway columnar epithelium hypertrophy (Figure 3). Lung tissue injury scores are shown in Figure 4.

### 3.3. Cell classification in BALF

**Figure 5:**
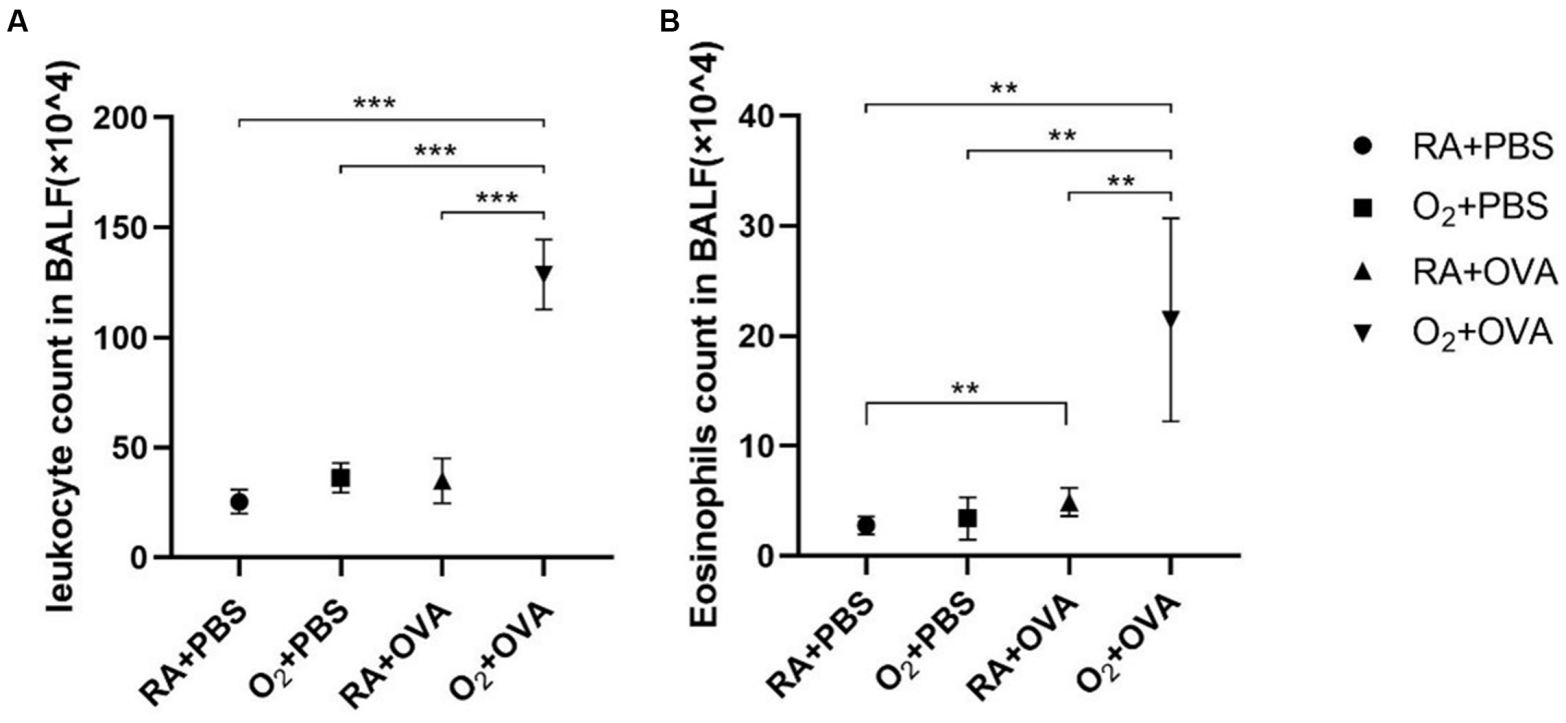
Airway inflammation in BALF samples (*n* = 8). **(A)** Leukocyte counts in BALF samples. **(B)** Eosinophil counts in BALF samples. ^***^p < 0.001. BALF: bronchoalveolar lavage fluid.

Total leukocyte count and eosinophil count were significantly higher in the BALF samples of mice in the O_2_ + OVA group than in those of the remaining three groups (*F* = 173.6; *p* < 0.05; Figures 5A,B).

### 3.4. Cytokine levels in BALF samples

#### 3.4.1. Th1 cytokine production in BALF samples

**Figure 6:**
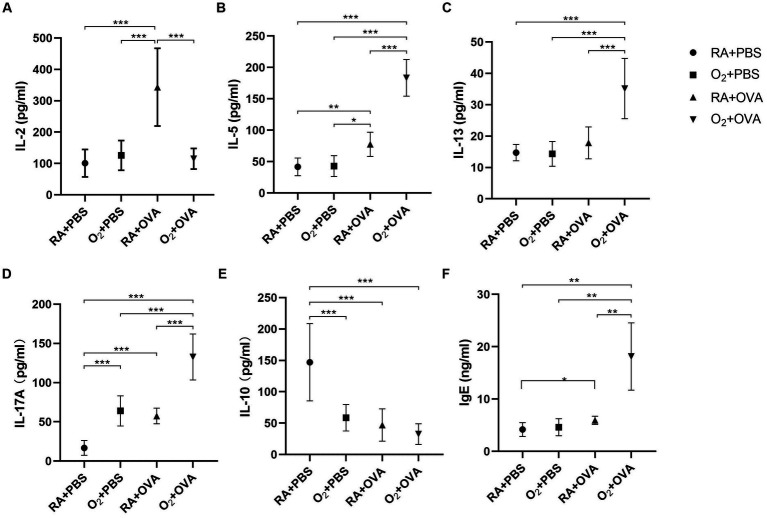
Cytokine levels in BALF samples (*n* = 8). Cytokiens levels of IL-2 **(A)** IL-5 **(B)** IL-13 **(C)** IL-17A **(D)** and IL-10 **(E)** in BALF samples. **(F)** Serum IgE level. ^*^ *p*<0.05, ^**^ *p* < 0.01, ^***^ *p* < 0.001. BALF, bronchoalveolar lavage fluid; IL, interleukin.

IL-2 levels were significantly lower in BALF samples of mice in the O_2_ + OVA group than in those of mice in the RA + OVA group (*p* < 0.05). No significant difference existed between the O_2_ + OVA group and the remaining two groups (*p* > 0.05; Figure 6A).

#### 3.4.2. Th2 cytokine production in BALF samples

IL-5 and IL-13 levels were significantly increased in BALF samples of mice in the O_2_ + OVA group. The BALF samples of mice in the O_2_ + OVA group had significantly higher IL-5 levels than those of mice in the remaining three groups (*p* < 0.05). The IL-5 levels were significantly higher in BALF samples of mice in the RA + OVA group than in those of mice in the RA + PBS and O_2_ + PBS groups (*p* < 0.05; Figure 6B). Mice in the O_2_ + OVA group had significantly higher IL-13 levels in BALF samples than those in the remaining three groups (*p* < 0.05). No significant differences existed among the other three groups (*p* > 0.05; Figure 6C).

#### 3.4.3. Th17, Treg cytokine production in BALF samples

IL-17A levels in BALF samples of mice in the O_2_ + OVA group were significantly higher than those of mice in the other three groups (*p* < 0.05; Figure 6D). IL-10 levels in the BALF samples of mice in the O_2_ + PBS, RA + OVA, and O_2_ + OVA groups were significantly reduced compared with those of mice in the RA + PBS group (*p* < 0.05). However, no significant differences existed among the O_2_ + PBS, RA + OVA, and O_2_ + OVA groups (*p* > 0.05; Figure 6E).

### 3.5. Serum IgE level

The serum IgE level was significantly higher in mice of the O_2_ + OVA group than in those of the other three groups (*p* < 0.01), and the serum IgE level in the room air+OVA group was higher than that in the room air+PBS group (*p* < 0.05; Figure 6F).

### 3.6. Lung microbiota sequencing

#### 3.6.1. OTU analysis

**Figure 7:**
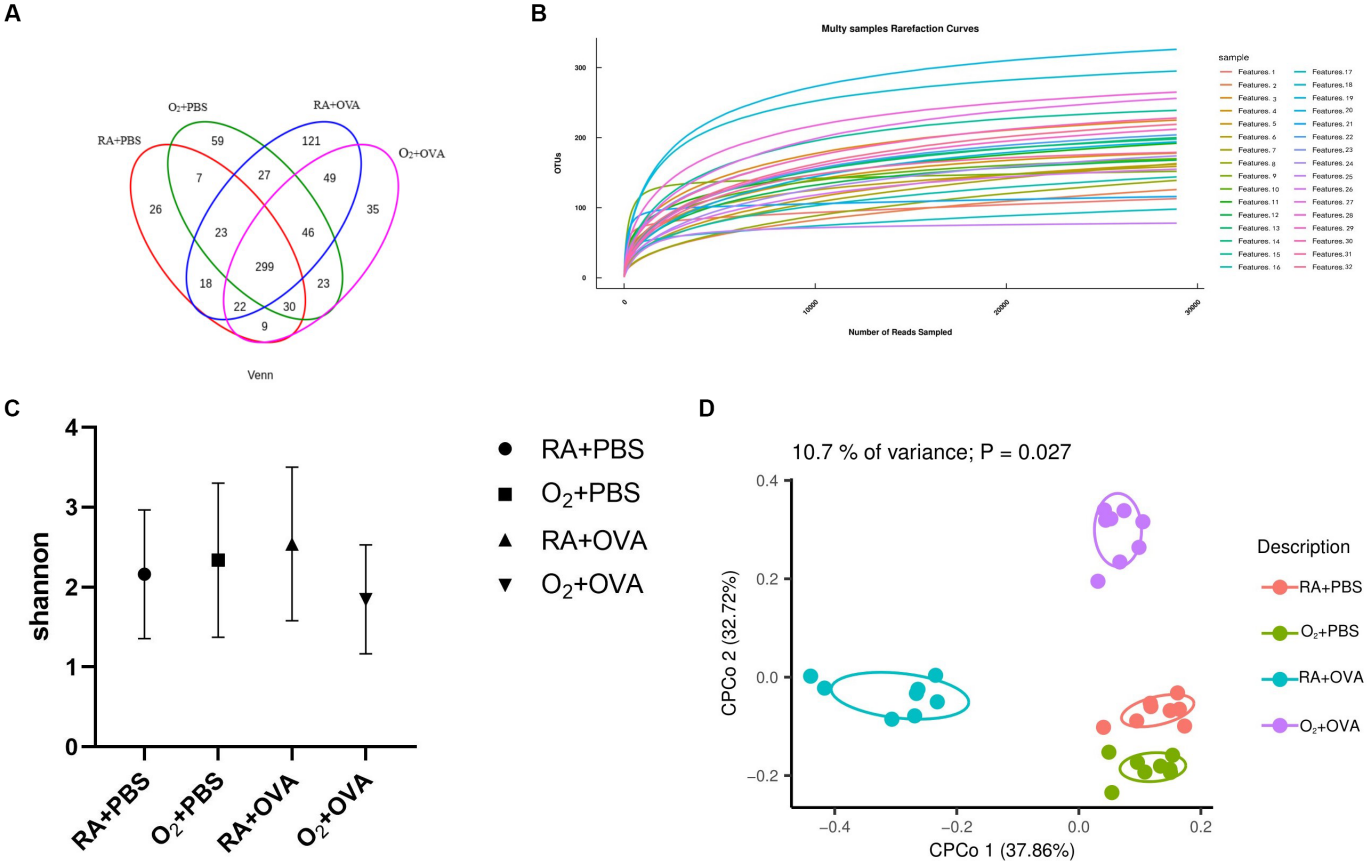
Analysis of pulmonary microbiota of the four groups. **(A)** Venn diagram of the pulmonary microbiota. **(B)** Rarefaction curve. **(C)** Alpha diversity analysis (using Shannon index) of the pulmonary microbiota (*p*>0.05); **(D)** CPCoA diagram of beta diversity of the pulmonary microbiota (*p*<0.05).

The OTU-based Venn diagram has four different colored circles that correspond to the lung microbiota of the four groups of mice. The overlap represents shared microflora between the groups. The pulmonary microflora was different between the groups (Figure 7A).

#### 3.6.2. Alpha diversity analysis

The alpha rarefaction curves of the mouse lung microbiota tended to be flat, which indicated that the sample sequencing data obtained in this study were gradually reasonable, and even though the data showed an increase, only a small number of new species would be generated (Figure 7B). The Shannon index of the lung microflora synthetically reflects the richness and evenness of the species, and the level of Shannon index is also affected by evenness, that is, the more homogeneous the species distribution in the samples, the higher the diversity is. No significant difference existed in the Shannon index among the four groups (*F* = 0.9297, *p*>0.05; Figure 7C).

#### 3.6.3. Beta diversity analysis

As shown in the CPCoA diagram, the four groups were clustered at different locations and were far from each other, with great diversity differences among the groups (Figure 7D).

#### 3.6.4. Microbial composition at the phylum and genus levels

**Figure 8:**
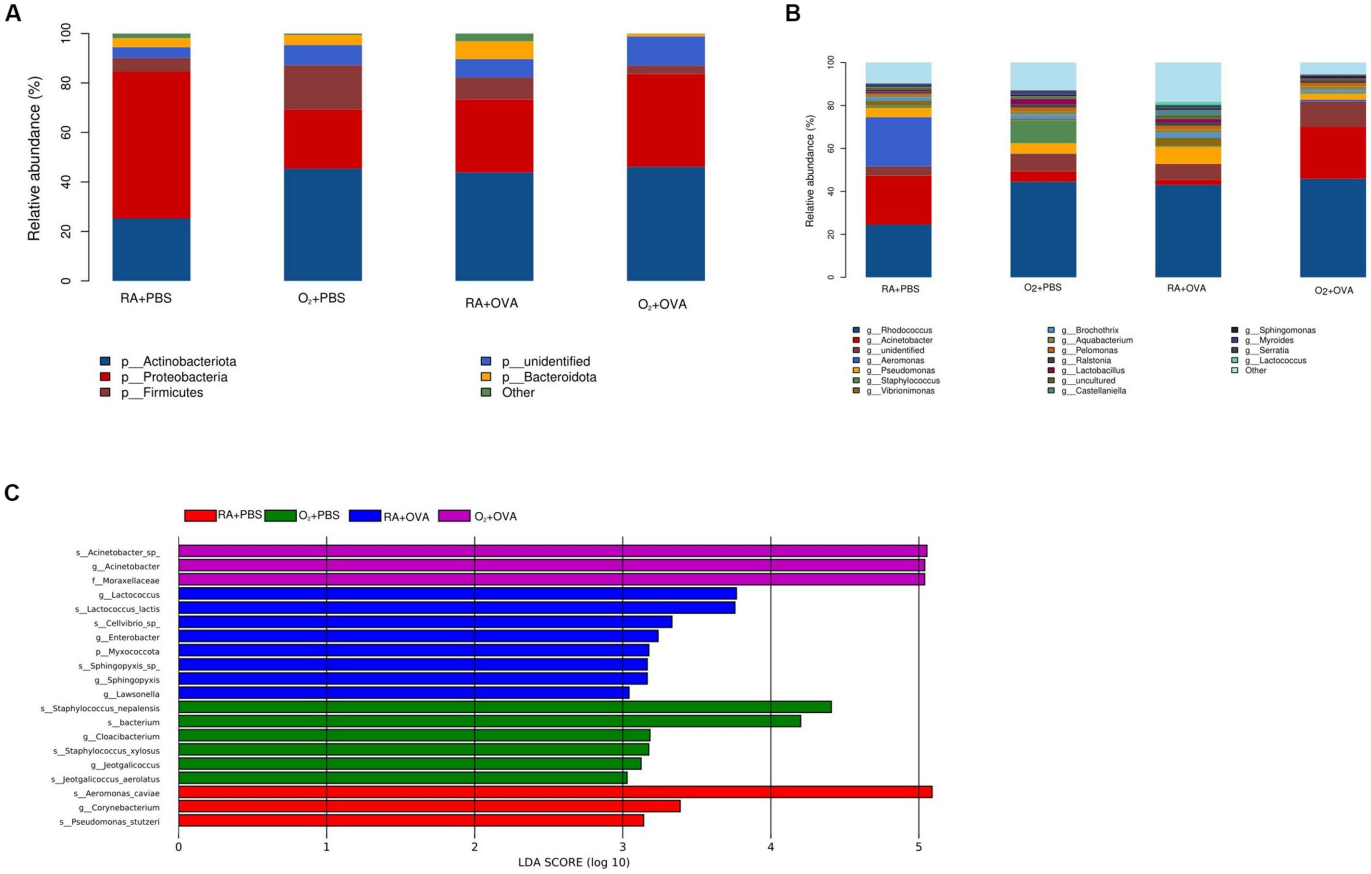
Microbial composition and relative abundance at the phylum and genus levels. **(A)** Composition of the lung microbiota at the phylum level. **(B)** Composition of the lung microbiota at the genus level. **(C)** LEfSe result. The histogram shows that colonies with LDA values >2 are statistically different biomarkers. When the default LDA value is more than 2 and the *p* value is less than 0.05. (phylum [P], class [C], order [O], family [F], genus [G], and species [S]).

Species classification of OTUs was performed by comparing databases and plotting histograms of the relative abundance of each species. At the phylum level, the main members of the pulmonary microflora were Actinobacteriota, Proteobacteria, Firmicutes, and Bacteroidota (Figure 8A). The main genera were *Rhodococcus*, *Acinetobacter*, *Aeromonas*, *Pseudomonas*, and *Staphylococcus* (Figure 8B).

#### 3.6.5. LEfSE analysis

The results of the LEfSE analysis showed that 20 bacterial microflora differed in terms of abundance in the lung tissues of the four groups. The bacteria with a major role were Aeromonas species (Aeromonas), Corynebacterium species, and Pseudomonas species (Pseudomonas) in the RA + PBS group; Staphylococcus species (Staphylococcus), Cloacibacterium species (Cloacibacterium), and Jeotgalicoccus species/genus (Jeotgalicoccus) are in the O_2_ + PBS group; Lactococcus species (Lactococcus), *Lactococcus lactis* species (*Lactococcus lactis*), Cellvibrio species (Cellvibrio), Enterobacter species (Enterobacter), Myxococcota phylum, Sphingomonas species/genus (Sphingopyxis), and Lawsonella genus (Lawsonella) are in the RA + OVA group; Acinetobacter species/genus (Acinetobacter) and Moraxellaceae family (Moraxellaceae) are in the O_2_ + OVA group (*p* < 0.05; Figure 8C).

## 4. Discussion

At birth, extremely preterm infants require respiratory support, and exposure to hyperoxia can cause neonatal lung injury and impede lung development, which eventually progresses to BPD (Thébaud et al., 2019). BPD can lead to short-term or long-term respiratory dysfunction. Some children with BPD have symptoms of periodic wheezing, which develop into childhood asthma (Fawke et al., 2010). Premature infants and children with BPD have a higher incidence of childhood asthma than children without a history of BPD (Di Fiore et al., 2019), and early hyperoxia exposure is an important perinatal factor that influences asthma development in children (Kim et al., 2018).

In the present study, mice of the O_2_ + OVA group showed evident asthma-like symptoms such as scratching the ears and gills, bending down, and wiping the nose. The pathological features of asthma were airway wall thickening, lumen stenosis, and airway columnar epithelial hyperplasia. Moreover, the counts of white blood cells and eosinophils and the levels of Th2 cytokines in BALF were increased, but the level of Th1 cytokines did not significantly change, suggesting that early hyperoxia exposure may enhance the ability of late OVA sensitization.

Increased secretion of type 2 cytokines is an important characteristic feature of asthma. Neonatal hyperoxia exposure promotes the development of asthma-like features including large infiltration of inflammatory cells around the bronchi, increased airway eosinophilia, and upregulated expression of the type 2 cytokines IL-5 and IL-13 (Cheon et al., 2018). Early hyperoxia exposure increases host susceptibility to allergen attack, which is reflected in the elevated levels of type 2 inflammation observed in the lungs after the OVA attack. Early life hyperoxia exposure enhances innate lymphoid cell (ILC2) function by increasing the expression of IL-33, and ILC2s produce type 2 cytokines such as IL-5, IL-13, and mediators involved in tissue repair, leading to eosinophilic airway inflammation and airway remodeling (Iijima et al., 2021). Antigen-presenting DC movement to lymph nodes and the activation of T cell responses have both been demonstrated to be facilitated by ILC2-derived IL-13 (Halim et al., 2014). The key mechanism by which early hyperoxia exposure functions is the production of reactive oxygen species (ROS). ROS can damage the airway epithelium and disrupt cellular function, thus leading to increased airway smooth muscle function, peri-airway extracellular matrix deposition in the lung interstitium, and cellular senescence, all of which affect the integrity of the airway epithelial barrier and result in airway remodeling (Parikh et al., 2019; Alva et al., 2022). Notably, neonatal hyperoxia exposure may not enhance the allergic response to OVA attack (Regal et al., 2014), possibly because of the differences in the timing of allergen sensitization and the duration of hyperoxia exposure. There is a “time window” for the effects of allergen exposure on asthma, with major changes being noted in the composition and function of the immune system during late birth and early childhood and a rapid influx of type 2 immune cells during the alveolar stage (PND4-21) when allergen sensitization predisposes to a type 2 immune response (de Kleer et al., 2016). In addition, early postnatal hyperoxia exposure leads to the large production of IL-5 and IL-13, which together are involved in asthmatic airway inflammation and airway remodeling effects (Cheon et al., 2018).

Ourteam also reported an increase in the levels of IL-17A cytokines and a decrease in the levels of IL-10 in BALF, and the Th17/Treg imbalance is an important mechanism in asthma development (Zheng et al., 2021). Hyperoxia significantly alters the microenvironment at the inflammation site, which promotes the polarization of Th17 and the secretion of cytokines such as IL-17A, which increase lung inflammation by recruiting neutrophils to the inflammation site (Nagato et al., 2015). IL-17A acts directly on bronchial smooth muscle cells, induces upregulated RhoA protein expression, triggers airway smooth muscle contraction and airway narrowing, and participates in AHR (Fong et al., 2018). Tregs mainly secrete anti-inflammatory factors such as transforming growth factor-β and IL-10, inhibit the activation and proliferation of effector T cells, suppress Th2 and Th17-mediated inflammatory responses, and prevent airway inflammation and bronchial hyperresponsiveness (Boonpiyathad et al., 2019). Zhao et al. demonstrated in a chronic airway inflammation model that Th17 cells suppress Treg-mediated tolerance (Hu et al., 2020) and that the Treg/Th17 imbalance is closely associated with asthma development and progression.

Hyperoxia exposure and OVA sensitization can exert an effect on the lung microflora. Hyperoxia exposure can alter the lung microbiota (Vieira et al., 2022), and dysbiosis of the lung microbiota can cause acute lung injury, wherein oxygen-induced changes in the lung microbiota precede the development of acute lung injury, and germ-free mice (mice without microorganism exposure) are protected from an oxygen-induced acute lung injury, suggesting that the deleterious effects of hyperoxia on lung injury are, at least partially, mediated by the lung microbiota (Ashley et al., 2020). Once established, the establishment of nonbeneficial respiratory microbiota triggers a self-reinforcing cycle of pro-inflammatory pathways that alter the respiratory microenvironment and may lead to long-term sequelae, including asthma and chronic obstructive (de Steenhuijsen Piters et al., 2020). The present study demonstrated that the abundance of *Staphylococcus aureus* was increased in the lung microflora of mice after hyperoxia exposure, and *Staphylococcus aureus* can regulate the immune response in the airway mucosa through its proteins, thereby inducing the activation of airway epithelial cells and the release of cytokines such as thymic stromal lymphopoietin, IL-25, and IL-33. This causes a sustained immune response in dendritic cells and ILC2 cells and the activation of type 2 immune response, thereby promoting the development of allergic airway disease (Chen et al., 2022). In addition, OVA sensitization alters the composition of the respiratory microbiota in mice, which may be associated with asthma development (Xiong et al., 2020; Zheng et al., 2021). Moreover, this study showed that *Acinetobacter* and Moraxellaceae were abundant in the O_2_ + OVA group, and were representative biomarkers of this group. One study reported low microbial richness in children with asthma and preschool children with wheezing and the predominance of the Moraxellaceae group. Moraxellaceae-dominant microflora caused a higher risk of asthma worsening as well as eosinophil activation, and *in vitro* experiments showed that Moraxellaceae induced epithelial cell damage and elevated the levels of the inflammatory factors IL-33 and IL-8 (McCauley et al., 2019; Chun et al., 2020). However, the significance and mechanism of action of alterations of the microflora are not fully understood.

## 5. Conclusion

In all, our study showed that the mouse suffered from postnatal hyperoxia exposure and early OVA sensitization in symptoms, respiratory pathology, alveolar lavage fluid cytokine levels, and lung microbiota during juvenile life, some of which behaved similarly to the classical asthma model. The findings of this study may provide a novel approach for the establishment of an asthma model using juvenile mice, and the detection of lung microbiota may also open new avenues for asthma research.

## Data availability statement

The original contributions presented in the study are included in the article/Supplementary material, further inquiries can be directed to the corresponding authors.

## Ethics statement

The animal study was reviewed and approved by the Ethics Committee of the Affiliated Huaian No. 1 People’s Hospital of Nanjing Medical University (DW-P-2021-002-01).

## Author contributions

ZT conceptualized and designed the study, and reviewed and revised the manuscript. JL and TB conducted the detection of flora. LC and MM conducted pathology and cytokine. YZ established the animal models. All authors contributed to the article and approved the submitted version.

## Funding

This study was financially supported by Key scientific research project of Jiangsu Provincial Health Commission (ZDB2020005) and Nanjing Medical University Science and Technology Development Fund (NJUB20210139).

## Conflict of interest

The authors declare that the research was conducted in the absence of any commercial or financial relationships that could be construed as a potential conflict of interest.

## Publisher’s note

All claims expressed in this article are solely those of the authors and do not necessarily represent those of their affiliated organizations, or those of the publisher, the editors and the reviewers. Any product that may be evaluated in this article, or claim that may be made by its manufacturer, is not guaranteed or endorsed by the publisher.

## References

[ref1] Akar-Ghibril N. Casale T. Custovic A. Phipatanakul W. (2020). Allergic Endotypes and phenotypes of asthma. J Allergy Clin Immunol Pract 8, 429–440. doi: 10.1016/j.jaip.2019.11.008 32037107 PMC7569362

[ref2] Alva R. Mirza M. Baiton A. Lazuran L. Samokysh L. Bobinski A.. (2022). Oxygen toxicity: cellular mechanisms in normobaric hyperoxia. Cell Biol. Toxicol. 39, 111–143. doi: 10.1007/s10565-022-09773-7, PMID: 36112262 PMC9483325

[ref3] Asher M. I. Rutter C. E. Bissell K. Chiang C. Y. El Sony A. Ellwood E.. (2021). Worldwide trends in the burden of asthma symptoms in school-aged children: global asthma network phase I cross-sectional study. Lancet 398, 1569–1580. doi: 10.1016/S0140-6736(21)01450-1, PMID: 34755626 PMC8573635

[ref4] Ashley S. L. Sjoding M. W. Popova A. P. Cui T. X. Hoostal M. J. Schmidt T. M.. (2020). Lung and gut microbiota are altered by hyperoxia and contribute to oxygen-induced lung injury in mice. Sci. Transl. Med. 12:eaau9959. doi: 10.1126/scitranslmed.aau9959, PMID: 32801143 PMC7732030

[ref5] Boonpiyathad T. Satitsuksanoa P. Akdis M. Akdis C. A. (2019). Il-10 producing T and B cells in allergy. Semin. Immunol. 44:101326. doi: 10.1016/j.smim.2019.101326, PMID: 31711770

[ref6] Chen H. Zhang J. He Y. Lv Z. Liang Z. Chen J.. (2022). Exploring the role of *staphylococcus aureus* in inflammatory diseases. Toxins 14:464. doi: 10.3390/toxins14070464, PMID: 35878202 PMC9318596

[ref7] Cheon I. S. Son Y. M. Jiang L. Goplen N. P. Kaplan M. H. Limper A. H.. (2018). Neonatal hyperoxia promotes asthma-like features through IL-33-dependent ILC2 responses. J. Allergy Clin. Immunol. 142, 1100–1112. doi: 10.1016/j.jaci.2017.11.025, PMID: 29253513 PMC6003836

[ref8] Chun Y. Do A. Grishina G. Grishin A. Fang G. Rose S.. (2020). Integrative study of the upper and lower airway microbiome and transcriptome in asthma. JCI Insight. 5:e133707. doi: 10.1172/jci.insight.133707, PMID: 32161195 PMC7141394

[ref9] Clemm H. H. Engeseth M. Vollsæter M. Kotecha S. Halvorsen T. (2018). Bronchial hyper-responsiveness after preterm birth. Paediatr. Respir. Rev. 26, 34–40. doi: 10.1016/j.prrv.2017.06.010 28709779

[ref10] de Kleer I. M. Kool M. de Bruijn M. J. Willart M. van Moorleghem J. Schuijs M. J.. (2016). Perinatal activation of the Interleukin-33 pathway promotes type 2 immunity in the developing lung. Immunity 45, 1285–1298. doi: 10.1016/j.immuni.2016.10.031, PMID: 27939673

[ref11] de Steenhuijsen Piters W. A. A. Binkowska J. Bogaert D. (2020). Early life microbiota and respiratory tract infections. Cell Host Microbe 28, 223–232. doi: 10.1016/j.chom.2020.07.004 32791114

[ref12] Di Filippo P. Dodi G. Ciarelli F. Di Pillo S. Chiarelli F. Attanasi M. (2022). Lifelong lung sequelae of prematurity. Int. J. Environ. Res. Public Health 19:5273. doi: 10.3390/ijerph19095273, PMID: 35564667 PMC9104309

[ref13] Di Fiore J. M. Dylag A. M. Honomichl R. D. Hibbs A. M. Martin R. J. Tatsuoka C.. (2019). Early inspired oxygen and intermittent hypoxemic events in extremely premature infants are associated with asthma medication use at 2 years of age. J. Perinatol. 39, 203–211. doi: 10.1038/s41372-018-0264-y, PMID: 30367103 PMC6351157

[ref14] Fang L. Zhou F. Wu F. Yan Y. He Z. Yuan X.. (2021). A mouse allergic asthma model induced by shrimp tropomyosin. Int. Immunopharmacol. 91:107289. doi: 10.1016/j.intimp.2020.107289, PMID: 33370683

[ref15] Fawke J. Lum S. Kirkby J. Hennessy E. Marlow N. Rowell V.. (2010). Lung function and respiratory symptoms at 11 years in children born extremely preterm: the EPICure study. Am. J. Respir. Crit. Care Med. 182, 237–245. doi: 10.1164/rccm.200912-1806OC, PMID: 20378729 PMC2913237

[ref16] Fong V. Hsu A. Wu E. Looney A. P. Ganesan P. Ren X.. (2018). Arhgef 12 drives IL17A-induced airway contractility and airway hyperresponsiveness in mice. JCI Insight 3:e123578. doi: 10.1172/jci.insight.123578, PMID: 30385725 PMC6238747

[ref17] Gollwitzer E. S. Saglani S. Trompette A. Yadava K. Sherburn R. McCoy K. D.. (2014). Lung microbiota promotes tolerance to allergens in neonates via PD-L1. Nat. Med. 20, 642–647. doi: 10.1038/nm.3568, PMID: 24813249

[ref18] Gough A. Linden M. Spence D. Patterson C. C. Halliday H. L. McGarvey L. P. (2014). Impaired lung function and health status in adult survivors of bronchopulmonary dysplasia. Eur. Respir. J. 43, 808–816. doi: 10.1183/09031936.00039513, PMID: 23900988

[ref19] Halim T. Y. Steer C. A. Mathä L. Gold M. J. Martinez-Gonzalez I. McNagny K. M.. (2014). Group 2 innate lymphoid cells are critical for the initiation of adaptive T helper 2 cell-mediated allergic lung inflammation. Immunity 40, 425–435. doi: 10.1016/j.immuni.2014.01.011, PMID: 24613091 PMC4210641

[ref20] Henderson W. R. Jr. Tang L. O. Chu S. J. Tsao S. M. Chiang G. K. Jones F.. (2002). A role for cysteinyl leukotrienes in airway remodeling in a mouse asthma model. Am. J. Respir. Crit. Care Med. 165, 108–116. doi: 10.1164/ajrccm.165.1.2105051, PMID: 11779739

[ref21] Herbst T. Sichelstiel A. Schär C. Yadava K. Bürki K. Cahenzli J. (2011). Dysregulation of allergic airway inflammation in the absence of microbial colonization. Am. J. Respir. Crit. Care Med. 184, 198–205. doi: 10.1164/rccm.201010-1574OC, PMID: 21471101

[ref22] Hou K. Wu Z. X. Chen X. Y. Wang J. Q. Zhang D. Xiao C.. (2022). Microbiota in health and diseases. Signal Transduct. Target. Ther. 7:135. doi: 10.1038/s41392-022-00974-4, PMID: 35461318 PMC9034083

[ref23] Hu Y. Chen Z. Zeng J. Zheng S. Sun L. Zhu L.. (2020). Th17/Treg imbalance is associated with reduced indoleamine 2, 3 dioxygenase activity in childhood allergic asthma. Allergy Asthma Clin. Immunol. 16:61. doi: 10.1186/s13223-020-00457-7, PMID: 32834826 PMC7386249

[ref24] Iijima K. Kobayashi T. Matsumoto K. Ohara K. Kita H. Drake L. Y. (2021). Transient IL-33 upregulation in neonatal mouse lung promotes acute but not chronic type 2 immune responses induced by allergen later in life. PloS One 16:e0252199. doi: 10.1371/journal.pone.0252199, PMID: 34048460 PMC8162637

[ref25] Kim A. Lim G. Oh I. Kim Y. Lee T. Lee J. (2018). Perinatal factors and the development of childhood asthma. Ann. Allergy Asthma Immunol. 120, 292–299. doi: 10.1016/j.anai.2017.12.009 29508716

[ref26] Mahmoud O. Granell R. Peralta G. P. Garcia-Aymerich J. Jarvis D. Henderson J.. (2023). Early-life and health behaviour influences on lung function in early adulthood. Eur. Respir. J. 61:2001316. doi: 10.1183/13993003.01316-2020, PMID: 36265880 PMC9978163

[ref27] Marathe S. J. Snider M. A. Flores-Torres A. S. Dubin P. J. Samarasinghe A. E. (2022). Human matters in asthma: considering the microbiome in pulmonary health. Front. Pharmacol. 13:1020133. doi: 10.3389/fphar.2022.1020133 36532717 PMC9755222

[ref28] McCauley K. Durack J. Valladares R. Fadrosh D. W. Lin D. L. Calatroni A.. (2019). Distinct nasal airway bacterial microbiotas differentially relate to exacerbation in pediatric patients with asthma. J. Allergy Clin. Immunol. 144, 1187–1197. doi: 10.1016/j.jaci.2019.05.035, PMID: 31201890 PMC6842413

[ref29] Nagato A. C. Bezerra F. S. Talvani A. Aarestrup B. J. Aarestrup F. M. (2015). Hyperoxia promotes polarization of the immune response in ovalbumin-induced airway inflammation, leading to a TH17 cell phenotype. Immun. Inflamm. Dis. 3, 321–337. doi: 10.1002/iid3.71, PMID: 26417446 PMC4578530

[ref30] Natalini J. G. Singh S. Segal L. N. (2023). The dynamic lung microbiome in health and disease. Nat. Rev. Microbiol. 21, 222–235. doi: 10.1038/s41579-022-00821-x, PMID: 36385637 PMC9668228

[ref31] Parikh P. Britt R. D. Jr. Manlove L. J. Wicher S. A. Roesler A. Ravix J.. (2019). Hyperoxia-induced cellular senescence in fetal airway smooth muscle cells. Am. J. Respir. Cell Mol. Biol. 61, 51–60. doi: 10.1165/rcmb.2018-0176OC, PMID: 30508396 PMC6604224

[ref32] Regal J. F. Lawrence B. P. Johnson A. C. Lojovich S. J. O'Reilly M. A. (2014). Neonatal oxygen exposure alters airway hyper-responsiveness but not the response to allergen challenge in adult mice. Pediatr. Allergy Immunol. 25, 180–186. doi: 10.1111/pai.12206, PMID: 24520985 PMC3976144

[ref33] Sánchez-García S. Rial M. J. Domínguez-Ortega J. (2020). Long and winding road: from infant wheeze to adult asthma. Curr. Opin. Pulm. Med. 26, 3–9. doi: 10.1097/MCP.0000000000000643 31688127

[ref34] Strachan D. P. (1989). Hay fever, hygiene, and household size. BMJ 299, 1259–1260. doi: 10.1136/bmj.299.6710.1259, PMID: 2513902 PMC1838109

[ref35] Sun T. Yu H. Y. Yang M. Song Y. F. Fu J. H. (2023). Risk of asthma in preterm infants with bronchopulmonary dysplasia: a systematic review and meta-analysis. World J. Pediatr. 19, 549–556. doi: 10.1007/s12519-023-00701-1, PMID: 36857022 PMC10198915

[ref36] Taylor S. L. Leong L. E. X. Choo J. M. Wesselingh S. Yang I. A. Upham J. W.. (2017). Inflammatory phenotypes in patients with severe asthma are associated with distinct airway microbiology. J. Allergy Clin. Immunol. 141, 94–103.e15. doi: 10.1016/j.jaci.2017.03.044, PMID: 28479329

[ref37] Thébaud B. Goss K. N. Laughon M. Whitsett J. A. Abman S. H. Steinhorn R. H.. (2019). Bronchopulmonary dysplasia. Nat. Rev. Dis. Primers. 5:78. doi: 10.1038/s41572-019-0127-7, PMID: 31727986 PMC6986462

[ref38] Vieira J. Jesudasen S. Bringhurst L. Sui H. Y. McIver L. Whiteson K.. (2022). Supplemental oxygen alters the airway microbiome in cystic fibrosis. mSystems 7:e0036422. doi: 10.1128/msystems.00364-22, PMID: 36000724 PMC9601246

[ref39] Wang W. Zhu H. Y. Tian Z. H. (2021). Effects of early postnatal hyperoxia exposure on ovalbumin-induced bronchial asthma model in mice. J. Nanjing Med. Univ. Sci. 41, 984–991. doi: 10.7655/NYDXBNS20210708

[ref40] Whiteside S. A. McGinniss J. E. Collman R. G. (2021). The lung microbiome: progress and promise. J. Clin. Invest. 131:e150473. doi: 10.1172/JCI150473, PMID: 34338230 PMC8321564

[ref41] Xiong Y. Hu S. Zhou H. Zeng H. He X. Huang D.. (2020). High-throughput 16S rDNA sequencing of the pulmonary microbiome of rats with allergic asthma. Genes Dis. 7, 272–282. doi: 10.1016/j.gendis.2019.03.006, PMID: 32215297 PMC7083718

[ref42] Zheng R. Wang F. Huang Y. Xiang Q. Dai H. Zhang W. (2021). Elevated Th17 cell frequencies and Th17/Treg ratio are associated with airway hyperresponsiveness in asthmatic children. J. Asthma 58, 707–716. doi: 10.1080/02770903.2020.1737710, PMID: 32114839

[ref43] Zheng J. Wu Q. Zou Y. Wang M. He L. Guo S.. (2021). Respiratory microbiota profiles associated with the progression from airway inflammation to remodeling in mice with OVA-induced asthma. Front. Microbiol. 12:723152. doi: 10.3389/fmicb.2021.723152, PMID: 34526979 PMC8435892

